# NMR and Metabolomics—A Roadmap for the Future

**DOI:** 10.3390/metabo12080678

**Published:** 2022-07-23

**Authors:** David S. Wishart, Leo L. Cheng, Valérie Copié, Arthur S. Edison, Hamid R. Eghbalnia, Jeffrey C. Hoch, Goncalo J. Gouveia, Wimal Pathmasiri, Robert Powers, Tracey B. Schock, Lloyd W. Sumner, Mario Uchimiya

**Affiliations:** 1 Departments of Biological Sciences and Computing Science, University of Alberta, Edmonton, AB T6G 2E9, Canada; 2 Department of Pathology, Department of Radiology, Massachusetts General Hospital, Harvard Medical School, Boston, MA 02114, USA; lcheng@mgh.harvard.edu; 3 Department of Chemistry and Biochemistry, Montana State University, Bozeman, MT 59715, USA; vcopie@montana.edu; 4 Complex Carbohydrate Research Center, University of Georgia, Athens, GA 30602, USA; aedison@uga.edu (A.S.E.); goncalog@uga.edu (G.J.G.); mario.uchimiya@uga.edu (M.U.); 5 Department of Biochemistry & Molecular Biology, University of Georgia, Athens, GA 30602-0001, USA; 6 Department of Molecular Biology and Biophysics, UConn Health, Farmington, CT 06030-3305, USA; heghbalnia@uchc.edu (H.R.E.); hoch@uchc.edu (J.C.H.); 7 Nutrition Research Institute, Department of Nutrition, School of Public Health, University of North Carolina at Chapel Hill, Chapel Hill, NC 27599, USA; wimal_pathmasiri@unc.edu; 8 Department of Chemistry, University of Nebraska-Lincoln, Lincoln, NE 68588-0304, USA; 9 Nebraska Center for Integrated Biomolecular Communication, University of Nebraska-Lincoln, Lincoln, NE 68588-0304, USA; 10 National Institute of Standards and Technology (NIST), Chemical Sciences Division, Charleston, SC 29412, USA; tracey.schock@nist.gov; 11 Interdisciplinary Plant Group, MU Metabolomics Center, Bond Life Sciences Center, Department of Biochemistry, University of Missouri, Columbia, MO 65211, USA

**Keywords:** NMR spectroscopy, metabolomics, review, advances, imaging

## Abstract

Metabolomics investigates global metabolic alterations associated with chemical, biological, physiological, or pathological processes. These metabolic changes are measured with various analytical platforms including liquid chromatography-mass spectrometry (LC-MS), gas chromatography-mass spectrometry (GC-MS) and nuclear magnetic resonance spectroscopy (NMR). While LC-MS methods are becoming increasingly popular in the field of metabolomics (accounting for more than 70% of published metabolomics studies to date), there are considerable benefits and advantages to NMR-based methods for metabolomic studies. In fact, according to PubMed, more than 926 papers on NMR-based metabolomics were published in 2021—the most ever published in a given year. This suggests that NMR-based metabolomics continues to grow and has plenty to offer to the scientific community. This perspective outlines the growing applications of NMR in metabolomics, highlights several recent advances in NMR technologies for metabolomics, and provides a roadmap for future advancements.

## 1. Introduction

The use of NMR for structure determination and the quantification of small molecules has a long history in successfully characterizing the chemical composition of biological systems. One of the earliest applications of NMR included the use of ^31^P and ^13^C NMR to monitor the energetic and redox status of cells and tissues [1,2,3]. While these studies demonstrated the value of NMR for metabolism studies, a renaissance occurred with the emergence of metabolomics [4], i.e., the broad range analysis of measurable small molecules in biological samples. From the onset of metabolomics as a scientific discipline, there has been a competitive focus on maximizing the number of metabolites detected or expanding the coverage of the metabolome [5]. As a result, LC-MS has become the most popular platform for metabolomics studies [6], but the field has also benefited from the unique strengths and advances in NMR technologies along with continued developments in computational tools to analyze complex metabolite mixtures [7]. 

Indeed, NMR spectroscopy still offers several unique advantages over other metabolomic platforms [8,9]. It is non-destructive, unbiased, easily quantifiable, requires little to no sample preparation, has no need for chemical derivatization, and is the “gold standard” for the identification of novel compounds. Furthermore, NMR is easily automatable and exceptionally reproducible, making automated high-throughput metabolomics studies much more feasible and reliable with NMR compared to LC-MS or GC-MS. In addition to these strengths, NMR is particularly amenable to detecting and characterizing compounds that can be challenging for LC-MS analysis, such as sugars, organic acids, alcohols, polyols, and other highly polar compounds. Unlike NMR, LC-MS is limited to detecting compounds that readily ionize, which is further diminished by ion suppression common to complex, heterogenous mixtures. Furthermore, NMR is highly amenable to metabolic flux and metabolic imaging studies, making it ideally suited for probing living cells, tissues, and organs. NMR has also become the preferred, clinically approved route to measure plasma lipoprotein and cholesterol classes. NMR-based in vivo metabolomics profiling also has the potential to be implemented in the clinic using magnetic resonance imaging (MRI) scanners, which are widely available in hospitals [10]. Advances in electronics, magnet shielding, and cryo-technology are making NMR instruments smaller, cheaper, easier to maintain, and more clinically compatible. Improvements in magnet technology are also leading to higher field strengths than previously possible. In short, NMR has the potential to transform the field of metabolomics, yet its potential has barely been tapped. We believe it is time to awaken the sleeping giant.

This perspective outlines some of the ways that this “awakening” is currently happening. It highlights how some of the most recent advancements in NMR technology are being used in metabolomics and how they are providing significant improvements over competing approaches. These innovations include enhancements in NMR automation, data acquisition speed, and hardware. They also include advances in techniques for NMR-based metabolite quantification, metabolite imaging, as well as metabolic flux and cholesterol measurements. Simply stated, this review is intended to provide a guide for how NMR can and should be used for metabolomics. It is also intended to serve as a roadmap for future advancements in this fast-developing field.

## 2. Automated NMR

Over the past few years, several software tools have been developed to facilitate automated NMR data processing and compound identification for metabolomics. NMR data processing typically combines Fourier transformation, phasing, baseline correction, solvent peak removal, and chemical shift referencing into a single automated or semi-automated pipeline. Automated or semi-automated compound identification typically involves fully computerized or computer-aided spectral deconvolution via spectral matching to a large library of reference NMR spectra [11]. Most of these automated or semi-automated tools have been designed for handling one-dimensional (1D) ^1^H NMR spectra. Several commercial programs, including Chenomx NMRSuite [12], FoodScreener [13,14], and B.I. QUANT [15,16], support both semi-automated NMR data processing as well as automated or semi-automated small molecule identification and quantification. The Chenomx NMRSuite and B.I. QUANT software have been specifically optimized for analyzing biofluids such as urine, plasma, or serum. On the other hand, FoodScreener has been optimized for analyzing food or beverages such as wine, juice, and honey. FoodScreener and B.I. QUANT are instrument- and vendor-specific (600 MHz-only, Bruker-only), while the Chenomx NMRSuite works with most NMR instruments and most field strengths.

In addition to these commercial programs, several freely available academic programs have been developed to semi-automate compound identification or quantification using NMR spectral datasets. These include Batman [17], AQuA [18], ASICS [19], ASICS 2.0 [19], and rDolphin [20]. However, these programs do not support automated data processing, which means a separate software package such as NMRPipe [21] or NMRFx [22] must be used to process the data prior to analysis. This highlights a common challenge in the metabolomics field, the need for multiple software tools to complete the entire data processing pipeline; however, MetaboAnalyst (https://www.metaboanalyst.ca, accessed on 21 July 2022) and MVAPACK (https://mvapack.unl.edu, accessed on 21 July 2022) [23] are striving to resolve this need. To further address this software multiplicity problem, two all-in-one software programs that have recently been introduced, Bayesil [24] and MagMet [25], support fully automated NMR. Both Bayesil and MagMet can perform fully automated data processing and spectral deconvolution of 1D ^1^H NMR spectra to identify and quantify upwards of 50 to 60 compounds in three to four minutes. Like the Chenomx NMRSuite, Bayesil and MagMet work with most NMR instrument models and field strengths but are limited to analyzing a specific biofluid type such as serum, plasma, or fecal water. MagMet is currently being developed to handle beverages and other food extracts. Both Bayesil (http://bayesil.ca/, accessed on 21 July 2022) and MagMet (http://magmet.ca, accessed on 21 July 2022) are freely accessible through web servers.

The development of automated spectral assignment and metabolite quantification algorithms represents one of the most important developments for NMR-based metabolomics. Automation massively increases throughput by a factor of 5–10 times over manual analysis, reduces the likelihood of spectra processing or fitting errors, improves compound identification and quantification accuracies, and significantly improves reproducibility across multiple platforms and between labs [26]. Under ideal circumstances, an NMR system that includes fully automated steps for sample loading, data collection and processing, spectral deconvolution and metabolite annotation can collect and process >100 samples a day on a single instrument. In terms of accuracy, the typical coefficient of variation (CV) for metabolite quantification with an automated NMR system is <5%, whereas it is often >20% for most LC-MS or GC-MS systems [26]. Automation also sets NMR-based metabolomics apart from other metabolomics platforms. LC-MS and GC-MS metabolomics platforms can only process 20 to 30% as many samples (over a 24 h period) as an automated NMR platform, primarily because of the significantly longer chromatography run-times. Furthermore, LC-MS and GC-MS require multiple manual sample preparation steps, lengthy periods of computer processing, and extensive manual data analysis. The tremendous advantages offered by an automated NMR system have already been realized in the fields of lipid and lipoprotein profiling.

## 3. NMR and Quantification

Metabolomics studies are aimed at capturing an accurate and unbiased representation of the intact metabolome from collected biospecimens. A particularly important feature of NMR-based metabolomics is its ability to provide highly accurate and reproducible quantification of metabolites from complex metabolite mixtures [27]. This quantification is commonly achieved by the judicious addition of a known amount of a chemical standard to the biospecimen of interest. Internal standards are a popular choice for quantitative NMR (qNMR) because of the simplicity of sample preparation and data collection. A compound with a unique and simple chemical structure such as sodium trimethylsilylpropanesulfonate (DSS) or sodium trimethylsilylpropionate (TSP) with a single chemical shift distinct from known metabolites is simply added to the biospecimen at a known concentration. A high level of accuracy and reproducibility can be easily achieved by comparing NMR peak heights with the added internal standard. In this manner, qNMR routinely achieves an accuracy and precision of less than 5%, an uncertainty of less than 0.5%, and a linear response over concentrations ranging from 10 μM to 1 M, with limits of detection as low as 1 μM. qNMR can also employ an external or an electronic reference, such as ERETIC [28], but an internal standard has an overall better performance, as external and electronic references require regular calibrations [29]. As discussed below, higher magnetic fields, advances in cryoprobe, microprobe or sub-microprobe technologies, along with novel pulse sequence designs continue to improve the sensitivity of NMR experiments and significantly decrease the lower limits of metabolite detection and quantification [30]. Additionally, advances in the algorithms and software used for metabolite deconvolution have improved quantification accuracy and have also greatly broadened metabolite coverage within complex biological samples [31,32,33,34,35].

It is important to remember that a 1D ^1^H NMR spectrum provides a single snapshot of all of the NMR-detectable compounds present in a biospecimen. Depending on the nature of the biospecimen (biological tissue, biofluid) or cell extract under study, and the focus of the study (e.g., polar, non-polar metabolites, lipids), the resulting spectrum may contain the combined signals from 100 or more metabolites. Thus, a primary obstacle to the routine application of qNMR to metabolomics is the high level of NMR signal overlap. This signal overlap can obscure the reliable measurement of peak intensities or the integration of NMR signals. 1D ^1^H NMR spectra can be deconvoluted or simplified by using several techniques including computational analysis, two-dimensional (2D) NMR experiments, [36] liquid chromatography [37] or by detecting other nuclei besides ^1^H [38]. Experimentally reducing the number NMR signals in a spectrum can be achieved by directly removing metabolites via liquid chromatography, or indirectly by using ^31^P or ^15^N NMR to select for phosphorus or nitrogen-containing compounds. However, if one uses chromatography to simplify spectra, the separation process may lead to unintended perturbations that are irrelevant to the biological questions of interest. This could potentially lead to unreliable or erroneous results, but the application of appropriate standards or reference material may mitigate this concern. Alternatively, overlapping NMR signals can be dispersed by increasing the spectral-width or by increasing the number of dimensions (going from 1D to 2D spectra). Increasing the NMR spectral width can be easily achieved by choosing an appropriate NMR nucleus. For example, the typical ^13^C chemical shift range of 200 ppm is approximately 20 times larger than an ^1^H NMR spectrum. It is important to remember that the direct quantification of 1D ^13^C or 2D NMR spectra requires additional calibrations since NMR signal intensities (peak heights) for these kinds of spectra are modulated by other factors such as differences in spin coupling constants, NOEs, relaxation times, and experimental parameters. Furthermore, acquiring 1D ^13^C (on samples that have not been enriched with ^13^C) or 2D NMR spectra can easily require hours or more of instrument time compared to seconds for a comparable 1D ^1^H NMR spectrum. However, specialized NMR probes optimized for ^13^C detection can make rapid data collection more feasible [39]. Recently, a variety of NMR pulse sequences such as heteronuclear single quantum coherence (HSQC), HSQC_o_ [40], Q-HSQC [41], QQ-HSQC [42], and quantitative perfected and pure shifted HSQC or QUIPU HSQC [43] have been developed to reduce peak variability arising from differences in coupling constants and other parameters. Similarly, non-uniform sampling and other rapid data acquisition schemes (i.e., fast HSQC) can dramatically reduce NMR acquisition times, making 2D NMR experiments practical for large metabolomics datasets. However, care must be taken when using such approaches for qNMR [44].

The use of multidimensional NMR, alternative NMR nuclei (e.g.,^13^C, ^15^N, and ^31^P), and solid-state NMR is expanding the capabilities of qNMR and holds considerable promise for NMR-based metabolomics. Despite the prospects and potential of qNMR for metabolomics [31], this approach has seen limited usage to date. Indeed, most published NMR metabolomics studies still rely on relative, instead of absolute, quantitative metabolite measurements. More widespread adoption of qNMR techniques by the NMR metabolomics community will be critical to making better use of the intrinsic advantages that NMR has over most other metabolomics platforms.

## 4. Metabolite Imaging, In Vivo NMR, and Clinical NMR

Different medical conditions present with distinct metabolic activities and metabolic abnormalities. For decades, metabolic alterations have been detectable using ex vivo medical NMR studies and in vivo magnetic resonance spectroscopy (MRS). The utility of MRS has led to its implementation in various clinical fields ranging from oncology to neurology. However, early clinical MRS and MRS imaging (MRSI) were limited because of low spectral resolution. This was due to the low magnetic field strength and relatively low field homogeneity of clinical MR scanners compared to ex vivo NMR instruments. Attempts to overcome these challenges have focused on improving both imaging hardware and data processing software. For instance, to enhance MR signal detection hardware, several specially designed surface coils, such as endorectal coils for prostate cancer or endovaginal coils for cervical cancer, have been developed [45,46]. Data processing software improvements have focused on enhancing the way that MRS images can be displayed. In contrast to univariate or intensity-based imaging data (such as X-rays or CT scans), MRS and MRSI data are multivariate (i.e., all measurable metabolites) and cannot be readily interpreted through simple visual evaluations. Instead, MRS and MRSI data interpretation must rely on computer assistance, artificial intelligence (AI) or machine learning. Several analytical software packages have been developed to analyze clinical MRS data and visualize their clinical implications. These include the widely used LCModel [47] and jMRUI [48] programs, which can automatically identify and quantify metabolites contributing to the signals seen in MRS and MRSI spectra. These software tools are very similar in concept to other software packages (such as MagMet, Bayesil and B.I. QUANT) used for automated ex vivo NMR metabolomics. Continuing developments in MR scanner technologies, including higher magnetic field strengths and improved coil array designs, have significantly increased our ability to generate MRS images with greater spatial resolution [49,50,51,52], as well as investigations of tissue cellular microstructures through diffusion-weighted MR spectroscopy [53]. Thanks to these improvements, MRS and MRSI are now offering metabolomics researchers and clinicians the ability to monitor detailed metabolic changes at high spatial resolution with good sensitivity in real time, in living organisms or in live patients. For example, NMR has been applied for identifying inborn errors of metabolism in clinical settings [16,54]. No other metabolomics technology (not LC-MS or GC-MS) offers this kind of chemical window on living systems. However, the use of MRS and MRSI in metabolomics studies has been remarkably light, and its promise remains largely unfulfilled. More widespread adoption of MRS and MRSI by the NMR metabolomics community will be key to bringing this technology into the mainstream of metabolomics studies.

Of course, the application of NMR metabolomics to clinical studies (i.e., diagnosis or prognosis of human diseases) is not limited to MRS and MRSI spectra. High-resolution NMR (i.e., traditional NMR metabolomics), which is routinely used for fundamental research, is of equal value to clinical research [55,56,57]. High-throughput 1D and 2D NMR experiments present several advantages to the clinician that include fast and reproducible data acquisition, low cost per sample, and minimal sample preparation or intervention.

## 5. Lipoprotein Profiling and NMR

Another important advance in NMR-based metabolomics has been the development of automated tools for lipid and lipoprotein particle (LDL, HDL, VLDL, etc.) analysis [58,59,60,61]. Lipoprotein particles are the metabolic by-products of cholesterol metabolism and consist both of proteins and cholesterol-containing lipids. As such, lipoprotein profiling is an important field of lipid metabolism and metabolomics. The first techniques for NMR-based lipoprotein analysis in serum/plasma were described in 1991 by Jim Otvos and colleagues [62]. Otvos showed how 1D ^1^H NMR spectra could be rapidly and automatically deconvoluted to identify lipoprotein components and extract accurate lipoprotein concentrations. This led the creation of LipoScience Inc. in 1994. LipoScience was initially dedicated to performing research on NMR-based cholesterol testing using plasma and serum samples. This effort eventually led to the development of a successful FDA-approved LDL test called LipoProfile in 2005 [63]. The LipoProfile NMR test is highly automated and provides 11 different measures of lipoprotein concentrations and sizes from plasma samples. In 2014, LipoScience was acquired by Labcorp, which now uses the same technology to offer comprehensive NMR-based lipoprotein profiling in many lab centers across the US and Canada. NMR-based lipoprotein profiling has become the “gold-standard” for lipoprotein measurement by clinicians because of its speed, accuracy, and the number of possible measurements [64,65]. Indeed, NMR-based lipoprotein profiling represents one of the more successful examples of metabolomics being translated into clinical practice.

Due to the success of LipoScience, several other companies, including Bruker (IVDr Lipoprotein Subclass Analysis (B.I. Lisa)) and Nightingale, have begun to offer lipoprotein analyses of serum and plasma samples through an automated NMR spectral data collection and fitting process [15,66]. These metabolomics profiling techniques are now available as either an on-site subscription-based service or an off-site clinical service. Both Bruker and Nightingale use spectral deconvolution or spectral fitting concepts such as the technique pioneered by LipoScience but offer more identified features or parameters. Bruker’s B.I. Lisa can measure 112 lipoprotein parameters [67], while Nightingale’s service reports on 228 lipoprotein parameters, including 20–30 small molecules [68]. Nightingale’s automated NMR pipeline is fast and inexpensive, which allows metabolomics to be performed at a scale unmatched by LC-MS, GC-MS, or CE-MS platforms. Indeed, Nightingale recently used its NMR platform to analyze >120,000 samples from the UK BioBank. Many large biobanks and research organizations are now turning to companies like Bruker and Nightingale to analyze tens of thousands of samples because of the high throughput, low cost, and broad metabolite coverage. The successful commercialization of these NMR-based metabolomics pipelines demonstrates the tremendous potential that NMR offers for future high-throughput metabolomics, lipidomics, and lipoprotein profiling. Based on the observed growth in medical testing and diagnostics, it is likely that NMR-based lipoprotein profiling will soon represent the majority of samples processed by the entire metabolomics community.

## 6. Fluxomics and In Situ NMR

**Figure 1:**
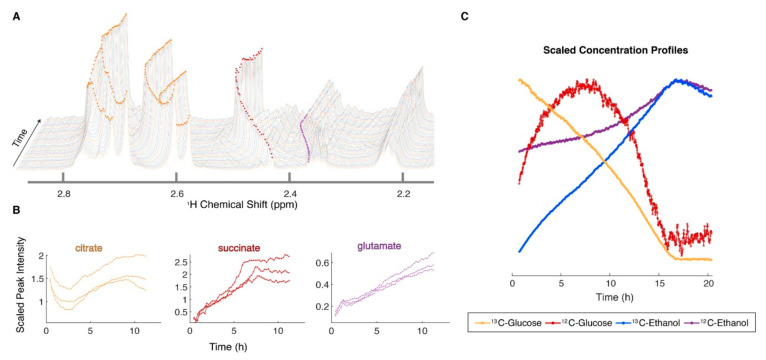
Continuous in vivo metabolism by NMR can be used to monitor the real-time growth of a microorganism under different environmental conditions. The data in (**A**) are from the filamentous fungus *Neurospora crassa*, growing in a high-resolution magic angle spinning probe at 600 MHz for about 12 h [10]. Oxygen can be introduced through a hole drilled into the cap of the NMR rotor [77]. The organism is alive at the end of the NMR experiment. The selected ridges shown in (**B**) were from 3 replicates and can be extracted from the NMR data using a computer vision algorithm [78] and plotted as a function of time. Isotopic substrates can also be used in this experiment (**C**), which allows for tracing of different pools of metabolites, as described more completely in Judge et al. [10]. Reprinted with permission from Ref. [10]. Copyright 2021 American Chemical Society.

Metabolomics routinely relies on an endpoint or final state measurement of a metabolic profile. Conversely, fluxomics studies the dynamic and temporal process of metabolite changes, metabolic reactions or metabolic fluxes [69]. As a result, metabolic reaction rates may be calculated from these measured fluxes. An important advantage of NMR for a fluxomics study is the fact that sample pre-preparation is not needed. This allows one to rapidly measure metabolic reactions in situ and on a real-time scale (Figure 1). NMR fluxomics studies have been conducted for decades through perfused measurements of animal organs using the injection of stable NMR-active isotopes (i.e., ^13^C- labeled compounds). Through these perfusion studies, a time-series of ^1^H, ^13^C, or ^31^P NMR spectra can be recorded, from which the intensities of the originally injected or perfused compounds and their reaction products can be quantified. The time-dependent series of peak intensities can then be used to produce reaction rates for all measurable and active metabolic pathways.

Fluxomics studies have been used in a wide range of pre-clinical and clinical metabolomics studies, including many involved in cancer [70,71,72,73]. Recent technology developments have further enhanced the use of fluxomics [74] by combining isotope labeling with hyperpolarized compounds. The use of hyperpolarized compounds and hyperpolarizing agents has resulted in upwards of a 1000× enhancement in NMR signals for certain compounds. Developments in high-resolution magic angle spinning (HRMAS) methods have also allowed for the mechanistic, real-time probing of cell-line metabolomics by similarly measuring isotope-labeled reactions [10]. This method has shown superior results in the fluxomic evaluation of aerobic metabolic pathways and in differentiating between intra- and extra-cellular metabolites.

While fluxomics studies are increasingly being performed using LC-MS methods coupled to isotopic perfusion techniques, it is important to remember that NMR-based fluxomics has a key advantage over LC-MS fluxomics. NMR-based fluxomics can exploit the ability of NMR to easily localize the exact position of a given (labeled) atom in a specific molecule. The spatial localization of specific ^13^C or ^2^H isotopes incorporated within a specific metabolite can provide a clear indication of the enzymes or pathways used to generate that metabolite [75,76]. Isotope labeling allows NMR-based fluxomics to easily link metabolites to proteins and pathways. In other words, NMR-based fluxomics offers a route to a complete, system-wide view of metabolism that is not achievable by almost any other method. Given the many strengths offered by NMR-based fluxomics, more widespread adoption of this approach by the NMR metabolomics community could lead to a closer link between metabolomics and systems biology.

## 7. Intact Tissue Metabolomics with HRMAS

NMR has a distinct advantage over techniques such as LC-MS for measuring metabolites in intact tissue. This is because LC-MS requires that one extract, homogenize and destroy tissues to measure their chemical composition. As a result, the tissue cannot be re-used or re-analyzed via microscopy by a pathologist. In contrast, living tissues or live biospecimens can be analyzed intact by NMR, with no need for extraction or homogenization. Indeed, NMR metabolomics studies of intact, living tissues have been conducted for many decades. However, the quality of NMR spectra collected from intact tissues tended to be quite poor, with relatively low spectral resolution and poor signal intensity. This was primarily due to tissue matrix effects leading to inhomogeneous signals and excessive line-broadening. Fortunately, these issues were resolved by the application of high-resolution magic angle spinning (HRMAS) to the analysis of intact tissues [79,80]. HRMAS involves the rapid spinning (6 to 10 kHz) of a sample at the magic angle (54.7°) to eliminate anisotropies and reduce the line-broadening effects arising from residual dipolar interactions and magnetic susceptibility variations.

High-resolution NMR spectra comparable to those measured from aqueous solutions can be obtained from biological tissues without any pre-treatment by using HRMAS. Furthermore, HRMAS does not destroy tissue architectures or alter the spatial location of metabolites. Thus, microscope-based pathological evaluations can be conducted on the same specimens after the HRMAS measurements have been completed. This unique capability of HRMAS was critical to the development of intact tissue metabolomics and to its adoption in several clinical settings or studies [81,82]. For instance, the presence and quantity of cancer lesions within a tissue sample analyzed by HRMAS would be unknown without a subsequent pathological evaluation of the tissues obtained from the suspected cancer patient. Accordingly, the conclusions drawn from HRMAS metabolomics studies can be clearly correlated with specific tissue pathologies. A further advantage of HRMAS NMR is its signal enhancement that allows for clinically informative metabolomics datasets to be measured on small tissue samples (<10 mg) [80], or a minute amount (<10 mL) of scarce human biofluid [83]. To better preserve tissue pathological architectures, various slow HRMAS methods have been proposed to ensure an accurate correlation between the metabolomics investigation and the disease pathology [80,84]. The ability of HRMAS NMR to characterize the metabolome of intact tissues non-destructively and quantitatively, coupled with its amenability to a post-analysis pathological or microscopic examination, makes HRMAS NMR an ideal tool for clinical metabolomics (especially biopsies) and metabolically guided anatomical studies. While both are still emerging areas of metabolomics, NMR and specifically HRMAS NMR are ideally suited to address these tasks.

## 8. NMR Techniques for Fast Data Acquisition

NMR-based metabolomics studies commonly rely on 1D ^1^H NMR spectral data that can be rapidly acquired in a few minutes with maximal signal to noise. However, 1D ^1^H NMR spectra tend to suffer from large solvent signals that obscure relevant peaks. They may also be affected by background signals arising from large biomolecules, as well as poor resolution and peak overlap due to a combination of limited spectral resolution and peak splitting from J-coupling. Several NMR pulse sequences have been developed to address each of these issues. For example, the first increment of a 2D nuclear Overhauser effect spectroscopy (NOESY) pulse sequence with pre-saturation (i.e., 1D NOESY), or pulse sequences that employ excitation sculpting or the PURGE pulse sequence, all provide efficient water suppression [55,85,86,87]. Similarly, the Carr-Purcell-Meiboom-Gill (CPMG) or PROJECT pulse sequence can efficiently remove background signals resulting from protein contamination using a T_2_ filter that relies on the large molecular-weight difference between small metabolites and large biomolecules [55,86,88]. A diffusion ordered spectroscopy (DOSY) edited pulse sequence can achieve a similar outcome using molecular weight-dependent differences in diffusion coefficients [89]. Of course, protein precipitation or protein filtering techniques may be a preferred alternative to removing protein contamination instead of relying on NMR pulse sequences [90]. The complexity of a 1D ^1^H NMR spectrum can be reduced by using isotopically (^13^C, ^15^N or ^2^H) labeled tracers or detecting alternative nuclei such as ^31^P [91]. Similarly, distributing the peaks into two dimensions can also reduce the spectral overlap and complexity. However, these 2D NMR approaches tend to result in substantially longer acquisition times (hours instead of minutes).

Fortunately, several recent discoveries and developments have occurred that can substantially reduce 2D acquisition times. For example, the use of non-uniform sampling (NUS) enables a more efficient acquisition of high-resolution 2D NMR spectra with significantly shorter acquisition times [92,93]. Instead of collecting the entire data matrix for a 2D NMR spectrum, NUS sub-samples only a fraction of the matrix, leading to a sparse data set. The resulting sparsity, usually 25–50%, directly determines the reduction in acquisition time. Other advancements in pulse sequences have led to further improvements in resolution and sensitivity [94]. For example, NUS and “pure shift” methods (see below) can be combined to yield an increased resolution along both dimensions in 2D experiments while still obtaining faster acquisition time. This is achievable because pure shift methods work independently of the chosen NUS schedule. In addition, several new pulse sequences have emerged for rapid acquisition of 2D NMR spectra, especially for ^13^C and ^15^N labeled samples (e.g., ASAP-HSQC, ALSOFAST-HSQC, CLIP-ASAP-HSQC, ASAP-/ALSOFAST-HSQC) [95]. NUS can be combined with these methods to achieve a further reduction in acquisition times with practically no loss in resolution and sensitivity [96]. Furthermore, Kupče et al. recently introduced the NMR by ordered acquisition (NOAH) super sequence using ^1^H-detection. NOAH combines two pulse sequence modules, ZZ-heteronuclear multiple bond correlation (HMBC) and ASAP-COSY, with multiplicity-edited HSQC and NOESY to obtain multiple NMR spectra from a single experimental measurement [87]. In essence, two or more NMR pulse sequences are interleaved and simultaneously acquired during the acquisition time of a single experiment. NMR, like numerous other analytical techniques, is highly dependent on state-of-the-art computers for data processing, analysis, and storage. The use of graphics processing units (GPUs) to advance and accelerate the application of artificial intelligence to challenging NMR problems is expected to transform NMR data processing. For example, a recent proof of principle application of deep neural networks has shown great promise in the reconstruction and processing of multi-dimensional NMR spectra acquired with NUS and sparse sampling while avoiding artifacts and distorted peak shapes and positions [97].

Other approaches have also emerged to improve resolution or shorten acquisition times for 1D NMR. In most NMR spectra, a significant reduction in resolution occurs due to the splitting of signals into multiplets resulting from J-coupling. “Pure shift” NMR spectroscopy is a broadband decoupling method that can be used to significantly enhance the resolution and sensitivity of an NMR spectrum by removing these splitting patterns [98,99,100]. Broadband homonuclear decoupling methods reduce multiplets to singlets through ^1^H-^1^H J-coupling removal, thereby reducing peak crowding, and improving resolution. These pure shift methods have been applied to both 1D ^1^H NMR spectra and to the indirect ^1^H dimension in 2D NMR experiments. Although the application of pure shift NMR, NUS and SOFAST methods to NMR metabolomics has been relatively minimal, the potential two-to-three-fold enhancement in signal sensitivity (via pure shift NMR) or the up to 10-fold faster data collection time (via SOFAST, ASAP or NUS methods) suggests that these methods should be routinely employed by the NMR metabolomics community.

## 9. Hardware Sensitivity Enhancement

Recent advances in NMR instrumentation have been aimed at lowering the traditional barriers to purchasing or using NMR spectrometers. In addition to developing turnkey instrumentation designed to streamline data collection and processing, the development of benchtop NMR spectrometers built using permanent magnets has led to a wealth of new applications that would have been logistically challenging with conventional NMR [69]. Benchtop NMR instruments (with operating frequencies ranging from 40 to 80 MHz) are less expensive and more compact and can be installed and employed at locations where NMR spectroscopy has not been practical due to physical or financial constraints. Additionally, the lower cost of operation for benchtop NMR spectrometers, which are built around permanent magnets as opposed to cryogenically cooled magnets, will enable NMR metabolomics to enter new underserved arenas that are inaccessible with current NMR technologies.

**Figure 2:**
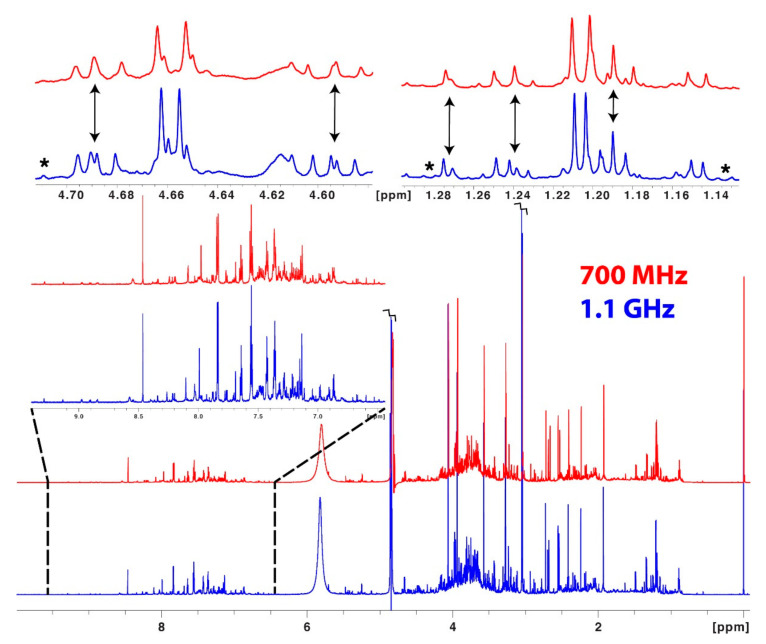
Expansions of experimental NMR data of the same sample of human urine collected in 5 mm tubes at 700 MHz (top red) and 1.1 GHz (bottom blue). For the 700 MHz data, the probe was a 5 mm quadruple resonance inverse CryoProbe (QCI-F). For the 1.1 GHz data, the probe was a 5 mm double resonance carbon-enhanced inverse (DCI) CryoProbe. The water suppression and baseline from the 1.1 GHz data are outstanding. This figure highlights some regions in which the increased chemical shift dispersion has resolved multiplets at 1.1 GHz compared to 700 MHz (indicated by arrows). There are also several small resonances that are difficult or impossible to recognize at 700 MHz that are clear at 1.1 GHz (indicated by *). Because the data were obtained with two types of probes and not fully relaxed, it is impossible to directly compare sensitivity gains across these datasets. Dr. Rainer Kuemmerle of Bruker BioSpin kindly provided the data.

NMR is the gold standard for establishing molecular connectivity and three-dimensional structures of small molecules. While its relatively lower sensitivity is often considered an Achilles’ heel, NMR sensitivity has significantly improved over the past couple of decades. The advent and advancement of very high field magnets have brought about significant improvements in sensitivity, which is non-linearly proportional to the magnetic field strength. Increased magnetic fields also provide improvements in spectral resolution and the simplification of scalar couplings, which can improve the accuracy of spectral deconvolution and thus also improve sensitivity. Most research-intensive universities have multiple high-field (e.g., 500 to 900 MHz) NMR instruments, while ultra-high field instruments (e.g., >900 MHz) are becoming accessible via shared resource centers. For example, the highest field NMR instrument, a 1.5 GHz instrument located at the USA National High Magnetic Field Laboratory (Tallahassee, FL, USA), is now available (~20 weeks, only short-duration experiments). Likewise, the National Science Foundation (NSF) has recently funded a 1.2 GHz instrument at Ohio State University and two NSF-funded 1.1 GHz instruments will be available through the Network for Advanced Nuclear Magnetic Resonance (NAN) and located at the University of Wisconsin, Madison and the University of Georgia, Athens. Despite the clear importance and growing need for ultra-high field instruments by the NMR community, the US lags in the acquisition of GHz instruments, whereas more than ten 1.2 GHz instruments have been delivered or are on order throughout Europe, including Florence (CERM), Italy, Zurich, Switzerland, Grenoble, France, and Utrecht, Netherlands. Many of these instruments are beginning to be used in metabolomics applications, as they often double the number of metabolites that can be detected and quantified relative to more conventional (500–600 MHz) magnets. Figure 2 provides a comparison of experimental data collected at 700 MHz and 1.1 GHz using the same sample of human urine. The increased spectral dispersion at 1.1 GHz resolves many overlapping regions where multiplet structures were ambiguous at 700 MHz. Spectra improve at higher field for several reasons: First, the coupling (in Hz) is independent of field strength, while the chemical shifts (in Hz) are proportional to field strength. Consequently, multiplets appear narrower and thus better resolved at higher field strength. The second very important effect is a reduction of strong coupling, which distorts resonances when the chemical shift difference between two coupled nuclei is close to the size of their coupling. Figure 2 also reveals several peaks observable at 1.1 GHz that are too small to interpret at 700 MHz. This is not surprising, because urine contains thousands of metabolites, many of which fall below the limits of detection in lower-field NMR. Thus, using GHz-class instruments for 1D metabolomics analysis will result in more quantified metabolites in a metabolomics study. Thus, to further enable advances in NMR-based metabolomics, we encourage US funding agencies to continue to support and prioritize the acquisition of GHz NMR instruments to increase their accessibility to the metabolomics community.

The NMR probe is a critical component of overall sensitivity and performance. In metabolomics applications, a balance between performance and standardization across multiple labs is an important consideration. For example, many routine metabolomic applications of biofluids use standard 5 mm room temperature probes [86]. These standard probes are simpler to optimize and will often yield more reproducible data for samples with relatively high dissolved salt concentrations, but this is not the case for all NMR probes. The development of cryogenically cooled probes has greatly enhanced NMR sensitivity [101]. Both liquid nitrogen and helium refrigeration-based cryoprobes are available and reduce electronic noise by lowering probe—and in some configurations, preamplifier—temperatures. Compared to high-field magnets, cryoprobes can provide sensitivity enhancements at lower costs. The combination of high-field magnets and optimized probes offers the greatest sensitivity improvements to NMR experiments. However, salt and dielectric effects increase with increasing frequencies and sample size. At field strengths greater than 900 MHz, a 5 mm cryogenic probe designed for ^1^H detection is not recommended for anything other than low dielectric organic solvents [102]. To help circumvent this problem, small volume cryo-microprobes at a diameter of 1.7 mm or 3 mm are also available. The mass sensitivity increases as the diameter of the probe decreases. Thus, these small volume probes offer outstanding performance for mass-limited samples, enabling the identification of nanomole quantities of metabolites with 2D NMR experiments [103,104,105]. Even smaller, room temperature 1 mm solenoid coils can be used for metabolite structure validation using microgram sample quantities [106,107]. Finally, both cryogenic technology and optimized coil materials, such as high-temperature superconducting material, can be combined to enhance ^13^C detection [39,108,109]. ^13^C detection does not suffer from the same salt effects that limit 5 mm ^1^H cryoprobes because ^13^C frequencies are only a quarter of ^1^H frequencies at the same field strength. As probe technology and high-field magnets continue to improve, the detection of ^13^C and perhaps other non-^1^H nuclei will likely grow in importance to metabolomics investigators.

Hyperpolarization techniques are other instrumental or hardware developments that offer impressive increases in sensitivity. Hyperpolarization uses a variety of well-known spin exchange or spin pumping methods to increase the nuclear spin polarization close to unity or 100%. This is well above the thermal-equilibrium levels normally encountered. Thus, hyperpolarization can result in a several orders of magnitude increase in sensitivity (i.e., S/N increase of >10,000) [110]. ^13^C hyperpolarization has been recently applied to cancer and plant metabolomics [111,112,113]. Other noteworthy developments with the potential to improve the sensitivity of NMR metabolomics include paramagnetic lensing to focus the B_1_ field [114] and detectors that employ nitrogen vacancy (NV) centers in diamond films [115]. While the high cost of hyperpolarizing equipment (especially dynamic nuclear polarization (DNP)) has generally been a significant barrier to its widespread adoption, recent advancements in low-cost hyperpolarization techniques such as para-hydrogen hyperpolarization [116] and SABRE-sheath hyperpolarization [117] suggest that hyperpolarization methods may soon be accessible to the wider NMR metabolomics community.

The coupling of NMR spectrometers with other types of instruments (i.e., hyphenated NMR) continues to offer new modalities for NMR-based metabolomics applications. Starting with LC-NMR around the 1970s, applications of hyphenated NMR approaches have grown steadily. A few hyphenated techniques such as LC-NMR, LC-circular dichroism (CD)-NMR, solid phase extraction (SPE)-NMR, and SPE-MS-NMR have found applications in natural product discovery, drug metabolism studies, drug impurity studies, herbal medicine, and the study of chiral compounds [118,119,120]. Accordingly, these approaches are directly applicable to metabolomics studies, especially regarding the identification of unknown metabolites. The development of automated SPE systems coupled with MS and NMR has enabled the automated purification of targeted analytes coupled with structure elucidation and/or validation by both MS and NMR [121]. The use of computer controlled SPE purification and the advanced sensitivity of high-field NMR with cryo-probes or cryo-microprobes have enabled the characterization of microgram quantities of materials, as noted above. Specific examples have included HPLC-UV-SPE-NMR [122,123], HPLC-MS/MS-SPE-NMR [106,124], and UHPLC-MS/MS-SPE-NMR [121,125]. These hyphenated technology ensembles reduce the traditionally lengthy and laborious metabolite discovery and identification process [126]. Overall, the use of hyphenated NMR is a growing trend, particularly when applied to the structure elucidation of metabolite mixtures. Over the coming years, it is expected that hyphenated NMR will become standard in many NMR metabolomics labs.

A challenge remaining for the wide-range adoption and employment of advanced NMR technologies and pulse sequences such as DNP, HR-MAS, pure shift NMR, and NUS is that these technologies often require significant training and knowledge of NMR spectroscopy. The NMR expertise requirement often limits the accessibility of these improved methods for non-NMR experts, although NMR software developers are working hard to make several of these methods accessible to a broad community of NMR users, including non-NMR specialists.

## 10. Databases and Software for Compound Identification

NMR continues to be the gold standard for chemical identification, and most chemistry and natural products journals require evidence of a new chemical’s presumptive structure with NMR spectral data showing its atomic or molecular connectivity. Many fundamental tools relating to NMR data processing and analysis for chemistry have been available through NMRbox [27]. For structure elucidation, NMR has historically required substantial interpretive expertise; however, many new tools are evolving that enable a larger user base for NMR metabolite identification. Several substantial NMR databases are now available that allow searching of 1D and 2D NMR data for individual metabolites. These include the Human Metabolome Database (HMDB) [127], the Madison-Qingdao Metabolomics Consortium Database (MQMCD), the Biological Magnetic Resonance Bank (BMRB) [128,129], and more recently, the Natural Product Magnetic Resonance Database (NP-MRD) [130]. We encourage all to contribute to these valuable community resources to help expand their utility. In addition, algorithms have been developed to create molecular networks from 2D NMR data, allowing chemical annotation from NMR data to extend into unknown, structurally similar metabolites [128,131]. Software tools with software-specific spectral databases are also arising to facilitate the identification of both polar and non-polar metabolites in mixtures via both 1D NMR (e.g., Chenomx NMR Suite, B.I. QUANT, MagMet, Bayesil) and 2D NMR (e.g., COLMAR) [11,132]. Although the quantity of data contained in today’s NMR databases for authentic compounds is growing, these spectral databases are expected to remain far from comprehensive in the foreseeable future. Fortunately, great strides are being made in the large-scale prediction of NMR spectra using quantum mechanical (QM) principles and machine learning (ML) [133]. Indeed, the NP-MRD is one of the first examples of an NMR database to contain tens of thousands of predicted NMR spectra of known compounds derived from state-of-the-art QM and ML techniques. We expect that these approaches will help fill the void of authentic data in the near future. However, these methods still require more authentic (experimentally acquired) data for further validation and for improving prediction accuracy. Thus, we again encourage data contributions from the public with an expected return on investment by expanding the accuracy and completeness of the predicted data content.

## 11. Conclusion and Future Directions

NMR-based metabolomics has been applied to nearly every scientific field, including biomedicine, biomarker discovery and medical diagnosis, drug discovery and development, environmental science, agriculture, nutrition, food science, plant science, renewable energy, and systems biology [8,30]. The continued growth of NMR-based metabolomics can be attributed to several unique qualities of NMR spectroscopy that are particularly valuable to the field of metabolomics. These include the fact that NMR spectra can be acquired rapidly and reproducibly, that NMR spectral properties are highly predictable and interpretable, and that NMR spectrometers are remarkably stable, long-lived, and very amenable to automation. In addition to these qualities, NMR has other compelling strengths. In particular, NMR requires little to no sample preparation, it preserves sample integrity, it supports accurate quantification, and it allows one to measure or image living samples and tissues. While these qualities are certainly known by members of the NMR metabolomics community, it is clear that these intrinsic strengths are not widely recognized by the broader metabolomics community.

The purpose of this review was to highlight several of the newest and most important developments in NMR and NMR-based metabolomics—developments that should put many of these criticisms to rest. A number of these innovations directly address the long-standing issues regarding NMR’s lack of sensitivity, its high cost, and its limited metabolite coverage. These include the development of ultra-high field NMR, including 1.1, 1.2 and even 1.5 GHz instruments (which potentially double the number of metabolites measurable by NMR), the development of low-cost hyperpolarization techniques (which can offer nanomolar detection limits), improvements in probe design (such as cryo-microprobes that give nanomole sensitivity), continued improvements in isotope tagging and isotope labeling (which extend metabolite coverage even further) and the development of new spectral deconvolution techniques in the area of lipidomics to detect hundreds of lipids and lipoprotein features.

Furthermore, as highlighted here, some of these developments extend the capabilities of NMR for metabolomics far beyond current practices in the metabolomics community. These developments include fully automated spectral processing along with automated metabolite identification and quantification (through tools such as B.I. QUANT and MagMet). They also include quantitative and automated cholesterol and lipoprotein profiling (via B.I. Lisa and other software), quantitative metabolite imaging through software programs such as LCModel and jMRUI, metabolic flux measurements with pathway tracing and enzyme attribution, HRMAS for intact tissue analysis, the development of novel pulse sequences and data acquisition methods (such as pure shift NMR, NUS, SOFAST) to greatly accelerate data collection, and the development of more hyphenated NMR systems (such as HPLC-UV-SPE-NMR, HPLC-MS/MS-SPE-NMR, and UHPLC-MS/MS-SPE-NMR). These advances are significant and, in many cases, will be truly transformative for the field of NMR metabolomics.

While recent developments herald exciting new capabilities unleashing new discoveries, change comes to the field slowly. The majority of recent published metabolomics studies continue to report the use of NMR methods and data analysis techniques that are 15–20 years old. In contrast, the MS-based metabolomics community has embraced new technologies (such as imaging mass spectrometry or ion mobility spectroscopy) and has pushed the envelope in terms of new methods or workflows to improve the performance of MS-based metabolomics. This rapid adoption of novel technologies has translated to significant advances in the field of MS-based metabolomics. A similar push to adopt emerging NMR technologies is needed to realize the full potential of NMR for solving important problems in metabolomics that remain refractory to MS-based methods or legacy NMR methods.

**Figure 3:**
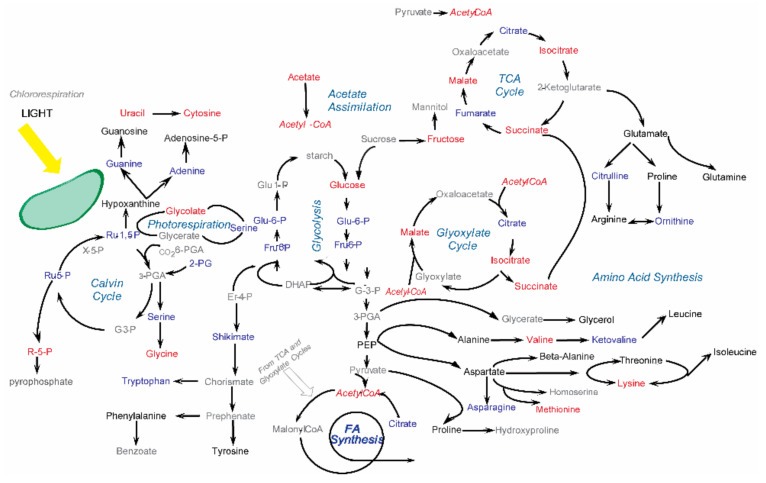
Metabolic pathway summarizing the compound-induced changes in the *C. reinhardtii* metabolome identified by NMR and GC-MS (metabolites of interest). Metabolites that were only identified by NMR are colored blue. Metabolites that were only identified by GC-MS are colored red. Metabolites identified by both methods are colored black, and metabolites not identified are colored grey. The total numbers of metabolites of interest within these metabolic pathways that were identified by either NMR, GC-MS, or both techniques were 14, 16 and 17 metabolites, respectively. Reprinted with permission from Ref. [134]. Copyright 2018 American Chemical Society.

Another imperative for advancing metabolomics is the need and opportunity to exploit the natural complementarity of NMR and MS. Neither method is capable of detecting *all* metabolites in a metabolomics sample. Instead of using NMR and MS independently, their combined usage improves the coverage of the metabolome and the accuracy of metabolite identification [134,135,136]. The best way forward is for the field to move beyond monolithic NMR-based or MS-based metabolomics studies and to embrace an integrative metabolomics protocol that employs multiple analytical techniques to maximize successful outcomes (Figure 3).

With recent advances in NMR, now is an opportune time for the broader metabolomics community to look more closely at the capabilities NMR has to offer to a metabolomics study. The bottom line is that NMR offers a treasure trove of tools, technologies, and techniques that can be more widely used by the metabolomics community. Metabolomics companies such as Nightingale and Olaris Therapeutics have realized this opportunity and have brought in hundreds of millions of dollars of investment. It is time for the rest of the NMR and metabolomics communities to take notice of modern NMR and integrative metabolomics and awaken this sleeping giant.
